# Regulation of cell proliferation and apoptosis in neuroblastoma cells by ccp1, a FGF2 downstream gene

**DOI:** 10.1186/1471-2407-10-657

**Published:** 2010-11-30

**Authors:** Francesca Pellicano, Rachel E Thomson, Gareth J Inman, Tomoko Iwata

**Affiliations:** 1 Paul O'Gorman Leukaemia Research Centre, University of Glasgow, Glasgow, UK; 2 School of Medicine, College of Medical, Veterinary and Life Sciences, University of Glasgow, Glasgow, UK; 3 Biomedical Research Institute, Ninewells Hospital and Medical School, University of Dundee, Dundee, UK

## Abstract

**Background:**

Coiled-coil domain containing 115 (Ccdc115) or coiled coil protein-1 (ccp1) was previously identified as a downstream gene of Fibroblast Growth Factor 2 (FGF2) highly expressed in embryonic and adult brain. However, its function has not been characterised to date. Here we hypothesized that ccp1 may be a downstream effecter of FGF2, promoting cell proliferation and protecting from apoptosis.

**Methods:**

Forced ccp1 expression in mouse embryonic fibroblast (MEF) and neuroblastoma SK-N-SH cell line, as well as down-regulation of ccp1 expression by siRNA in NIH3T3, was used to characterize the role of ccp1.

**Results:**

Ccp1 over-expression increased cell proliferation, whereas down-regulation of ccp1 expression reduced it. Ccp1 was able to increase cell proliferation in the absence of serum. Furthermore, ccp1 reduced apoptosis upon withdrawal of serum in SK-N-SH. The mitogen-activated protein kinase (MAPK) or ERK Kinase (MEK) inhibitor, U0126, only partially inhibited the ccp1-dependent BrdU incorporation, indicating that other signaling pathway may be involved in ccp1-induced cell proliferation. Induction of Sprouty (SPRY) upon FGF2 treatment was accelerated in ccp1 over-expressing cells.

**Conclusions:**

All together, the results showed that ccp1 regulates cell number by promoting proliferation and suppressing cell death. FGF2 was shown to enhance the effects of ccp1, however, it is likely that other mitogenic factors present in the serum can also enhance the effects. Whether these effects are mediated by FGF2 influencing the ccp1 function or by increasing the ccp1 expression level is still unclear. At least some of the proliferative regulation by ccp1 is mediated by MAPK, however other signaling pathways are likely to be involved.

## Background

A previously uncharacterized gene named Coiled-coil domain containing 115 (Ccdc115) or coiled coil protein 1 (ccp1) (GeneID: **69668**), has been recently identified downstream of Fibroblast Growth Factor 2 (FGF2) by microarray analysis and its expression pattern was characterized [1]. The ccp1 transcript was up-regulated upon FGF2 stimulation in primary cortical neuron culture (CNC) derived from mouse embryonic telencephalon at embryonic day 14.5 (E14.5) and in neuroblastoma cell line, SK-N-SH. In situ hybridizations revealed that ccp1 was expressed in the ventricular zone (VZ), a region of the developing cerebral cortex known to be composed of progenitor cells undergoing proliferation [2].

The mechanism by which cell proliferation is controlled in the VZ is still not fully understood. A number of factors, including FGFs, have been shown to regulate the proliferation of progenitor cells in embryonic Central Nervous System (CNS) in vitro [3-7]. FGFs are a family of 22 polypeptides known to play various roles in neural development [8,9]. FGF signals are mainly mediated by high-affinity receptor-type tyrosine kinases, FGF receptors (FGFRs). FGF signaling plays variety of roles in neural development and in pathogenesis of developmental diseases. FGFs are a class of molecules that regulate proliferation by controlling the length of the G1 phase. Addition of FGF2 in primary culture prepared from developing cortex at E14-E16 showed shortening of the G1 length and increase in proliferative divisions, indicating that FGF2 controls cell proliferation via its control of G1 length [10]. Regulation of cell proliferation is mediated by a complex system of signaling pathways. One of the core pathways downstream of FGF is the mitogen-activated protein kinase (MAPK) pathway [11], which has a central role in transmitting cell proliferation and survival signals [12]. In this pathway, RAS promotes activation of the serine/threonine protein kinases Raf1 and MEK1. In addition to controlling RAF kinases, MAPK may also directly regulate several other signaling pathway, such as the phosphatidylinositol 3 (PI3) kinase [13].

In this study, ccp1 function was investigated using a retroviral over-expression system and RNA interference (RNAi) in vitro. We analysed the effects of altered ccp1 expression in cell proliferation and apoptosis in mouse embryonic fibroblast (MEF), a neuroblastoma cell line SK-N-SH, and in NIH3T3. Since ccp1 expression is found regulated by FGF2, we also investigated the effects of FGF2 and compared them to those of the serum in ccp1-expressing cells. Furthermore, by specifically inhibiting the MAPK pathway with the pharmacological inhibitor U0126, we further investigated the involvement of this pathway in ccp1-induced cell proliferation. Our results showed that ccp1 regulates cell number by promoting proliferation and suppressing cell death.

## Methods

### Cell culture

SK-N-SH and MEF cells were maintained in D-MEM, 10% fetal bovine serum (FBS) and 2 mM glutamine. NIH3T3 were maintained in D-MEM, 10% DCS and 2 mM glutamine. The natural immortalised MEFs were originally from Dr Nick Dyson (Massachusetts General Hospital Cancer Center/Harvard Medical School, Charlestown, MA [14]. Cells were maintained at 37°C in 5% CO2. When required, cells were starved in media without serum for 24 h and treated with FGF2 and heparin as indicated.

### Retroviral-mediated expression of ccp1

Single strand cDNA was synthesized as described in [1]. The retrovirus expression vector pLPC was obtained from Dr S. Lowe. Phoenix packaging cells were transfected with pLPC/eGFP or pLPC-ccp1/eGFP-N vectors using Lipofectamine 2000 (Invitrogen). Cells were incubated ON in 20% fetal bovine serum (FBS) media in order to allow virus production. Immortalized MEF and SK-N-SH cells were infected with the Phoenix-supernatant. The infection was repeated three times at intervals of 12 h each. After the last infection, cells were selected in the presence of 25 mg/ml of puromycin.

### RNA interference (RNAi)

Transfection was performed using Lipofectamine 2000 reagent in Optimem media (Gibco) according to the manufacturer's instructions (Invitrogen). 50 nM/well of pre-designed and annealed siRNAs (Ambion) were used: siRNAi1, sense 5'-aguugaagccuuugacuuctt-3', anti sense 5'-gaagucaaaggcuucaacutc-3'; siRNAi2, sense 5'-ggcaugaaguugaguuaugtt-3', anti sense 5'- cauaacucaacuucaugcctc-3'. Scramble siRNA was purchased from Ambion. After 24 h, cells were harvested and RNA was extracted and used for semi-quantitative RT-PCR. The primers used were F-338 and R-1096 [1]. Primers were designated against a DNA sequence with very high homology between mouse and human DNA.

### BrdU assays

BrdU assay was performed using BrdU labeling and detection Kit I (Roche). Cells were exposed to BrdU for 1 h and fixed in ethanol for 20 minutes (min) at -20°C. Anti-BrdU antibody was applied for 30 min at 37°C, and the fluorescein-conjugated secondary antibody for 30 min. Coverslips were mounted with 4',6 diamidino-2-phenylindole (DAPI). Photographs were taken using a Zeiss Axioskop microscope and Axiovision software.

### Western blotting

Cells were lysed in Laemmli sample buffer (Biorad) and analysed on 12% SDS-PAGE. The proteins transferred to Hybond ECL nitrocellulose membranes (Amersham) were blocked with 10% dried milk in TBST (20 mM Tris, pH 7.6, 13.7 mM NaCl, 0.1% Tween 20) for 2 h. Incubation with the primary antibody was at 4°C overnight, and with the secondary, for 1 h at room temperature. Detection was with ECL (Amersham) exposed to X-ray film (Fuji). Antibodies were anti-ccp1 (1:500; anti-rabbit and anti mouse; Beatson Laboratories Antibody Services), anti-ERK, anti-p-ERK (Cell signaling), anti-Sprouty (Invitrogen) and anti-GFP (Abcam). Densitometry analysis was carried out by Quantity One program (Biorad).

### Tunel

TUNEL assays were performed using the In situ Cell Death Kit-AP (Roche). Cells were serum starved overnight and then fixed in 4% paraformaldehyde for 1 h at -20°C and permeabilised in 0.1% Triton X-100, 0.1% sodium citrate for 2 min at 4°C. The DNA strand breaks were fluorescently labeled via the TUNEL reaction for 1 h at 37°C. TUNEL-positive cells were detected by fluorescence (FITC, 520 nm).

### Statistics

Student's t-test was performed to test the significance of difference in numerical data as appropriate.

### Ethics

Our research conformed to the Helsinki Declaration and to local legislation.

## Results

### Morphological changes of the cell upon ccp1 over-expression

**Figure 1:**
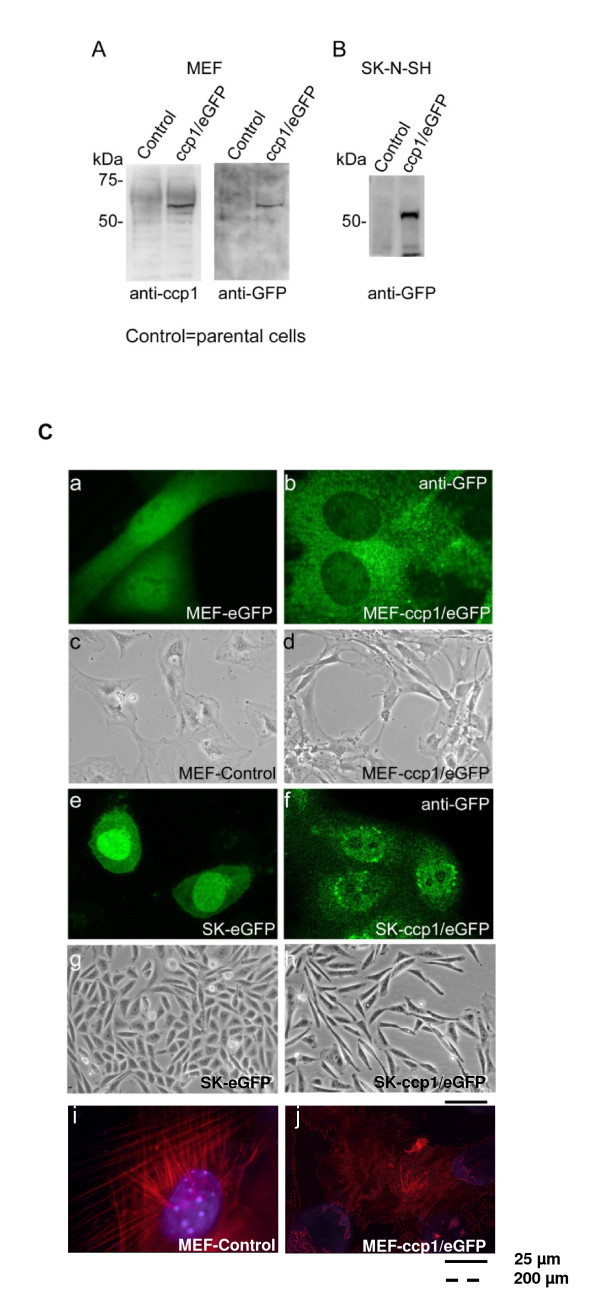
**Establishment of cell lines stably expressing the ccp1 protein**. Cell lines stably expressing ccp1/eGFP fusion proteins were generated using retroviral approach. (A) The expression of ccp1/eGFP fusion protein was analyzed in MEF cell lines using polyclonal sera raised against ccp1 (left panel) and GFP antibody (right panel). The ccp1/eGFP fusion protein was detected as a single band at the expected molecular weight of 55 kDa. (B) The expression of ccp1/eGFP fusion protein was also analyzed in neuroblastoma, SK-N-SH cell lines using GFP antibody. 7.5% SDS-PAGE. (C) MEF (a-d, i-j) and SK-S-NH (e-h) were cultured in the presence of serum. (a-b, e-f) Cells were analyzed using anti-GFP antibody and confocal microscopy. (a) Mostly nuclear and weak cytoplasmic localization was diffusely observed in the eGFP protein stably expressed in MEF. (b) The ccp1/eGFP fusion protein stably expressed in MEF was observed in the cytoplasm in punctate spots. (c) Control MEF presented a typical fibroblast flat shape. (d) MEF expressing ccp1/eGFP fusion protein appeared smaller and shaped spindle-like. (e-h) Similar pattern was observed in SK-N-SH cells. (i-j) Filamentous actin was visualized with Teas Red-conjugated phalloidin (red) in MEF control and MEF-ccp1/eGFP. Nuclei were stained using DAPI (blu). Scale bar in a-b, e-f, i-j = 25 μm (line) and in c-d, g-h = 200 μm (dotted line).

We first established MEF cells stably expressing ccp1 tagged with eGFP at the N-terminus (ccp1/eGFP-N) by retrovirus approach. The ccp1/eGFP expression vector was generated by using the retroviral vector pLPC. Expression levels of ccp1/eGFP-N fusion protein was detected under the phase contrast microscope and then analysed by Western Blotting (Figure 1A). The cells stably expressing ccp1/eGFP-N showed a band of 55 kDa, corresponding to the expected size of the ccp1/eGFP-N fusion protein which was detected either with an anti-GFP and the ccp1-specific antibody produced in house. We have previously shown that ccp1 is highly expressed in embryonic and adult brains and up-regulated in SK-N-SH cells after treatment with FGF2 [1]. Therefore we generated stably expressing cell line of ccp1/eGFP-N in human SK-N-SH neuroblastoma cell lines (Figure 1B). A specific band of 55 kDa was also observed in this stable line.

In order to identify cellular localization of the ccp1 protein, analysis with confocal microscopy was carried out in MEF and SK-N-SH stably expressing ccp1/eGFP-N protein using an anti-GFP antibody (Figure 1C, a-b, e-f). While control cells expressing eGFP showed only diffuse fluorescence signals throughout the cells, the ccp1/eGFP-N protein was observed in a localized and punctate pattern. Although ccp1 was mainly present in the cytoplasm in MEF cells, SK-N-SH cells showed expression also in the nucleus and peri-nuclear region as observed in primary CNC [1].

Signaling through FGF/FGFR is known to result in morphological transformation of fibroblasts in vitro, which may be associated with tumor progression [15,16]. Morphological transformation of the cell was observed upon stable expression of ccp1 in MEF and SK-N-SH (Figure 1C, c-d, g-h). Cells expressing ccp1/eGFP-N appeared smaller and had a spindle-like phenotype compared to the control cells. Morphological transformation in MEF stably expressing ccp1/eGFP-N protein was also shown by cytoskeletal staining of the actin filaments, in which actin cytoskeleton actin cytoskeleton actin cytoskeletonMembrane ruffling is visualized by staining the actin cytoskeleton actin remodelling and membrane ruffling was indicated upon ccp1 over-expression (Figure 1C, i-j).

### Stable expression of ccp1 leads to an increase in cell number

**Figure 2:**
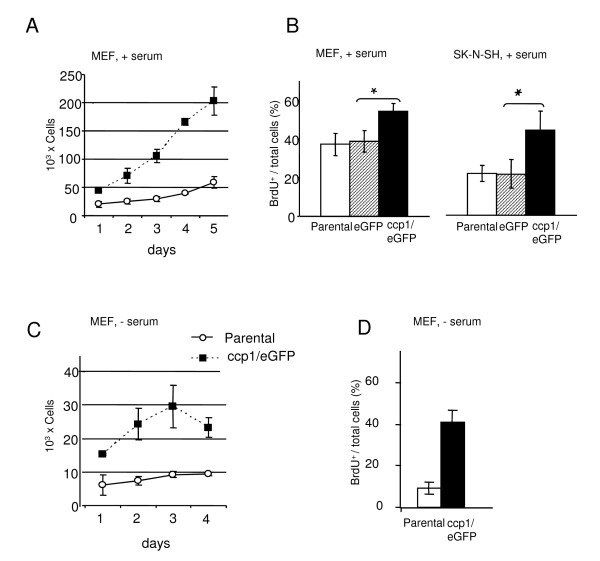
**Stable expression of the ccp1 protein resulted in an increase in cell number**. (A) Parental MEF and MEF expressing ccp1/EGFP fusion protein were cultured in the presence of serum over 5 days. (B) MEF expressing the ccp1/eGFP protein showed increase in BrdU incorporation in comparison to control cells. BrdU incorporation was also examined in SK-N-SH cells. Cells expressing the ccp1/eGFP protein showed an increase in BrdU incorporation in comparison to control. (* p < 0.05) (n = 3). (C) In the absence of serum, 3-fold increase in cell number was observed in MEF stably expressing ccp1/eGFP fusion protein in comparison to parental MEF at 3 days of culture. The number of cells expressing ccp1/eGFP decreased afterwards. (D) MEF expressing the ccp1/eGFP protein showed increase in BrdU incorporation in comparison to control cells also when cultured in absence of serum. Scale bar = 200 μm.

Next, in order to determine the function of ccp1 in cell proliferation, a growth of cells stably expressing ccp1 was examined in the presence of serum for up to 5 days (Figure 2). MEF stably expressing ccp1 showed 123% increase in cell number in one day in culture (Figure 2A). After 5 days, MEF expressing ccp1 grew 4-fold more than control, indicating that stable over-expression of ccp1 promoted cell proliferation. To determine whether the observed increase in cell number was due an increase in cells in the S phase, BrdU assay was performed in MEF and SK-N-SH cells stably expressing ccp1/eGFP-N cultured in the presence of serum (Figure 2B). Cells were cultured on coverslips in 10% serum for 24 h, incubated in 10 μM BrdU for 1 h before fixation, and stained with anti-BrdU antibody. MEF expressing ccp1 showed an increase in the number of cells in the S phase by 45% compared to that in control parental MEF and to MEF expressing eGFP. Similar results were obtained in SK-N-SH cells expressing ccp1, which showed an increase in cell proliferation by two-fold compared to the control and to the SK-N-SH stably expressing eGFP.

In order to determine whether ccp1 could actually induce cell proliferation independent of growth factor stimulations, growth of cells were examined in MEF expressing ccp1/eGFP-N in the absence of serum (Figure 2C). Control MEF showed a mild increase in cell number for up to 4 days. In contrast, MEF stably expressing ccp1/eGFP-N showed a three-fold increase in cell number at 3 days in culture and then a decrease was observed on the 4^th ^day. Similarly, BrdU assay was performed in MEF cells stably expressing ccp1/eGFP-N and cultured in the absence of serum (Figure 2D). As expected control parental MEF showed a marked decrease in BrdU incorporation when compared to cells grown in the presence of serum (Figure 2B and 2D). Interestingly, however, MEF expressing ccp1 showed an obvious increase in the number of cells in the S phase compared to that in control parental MEF even in the absence of serum.

These data indicated that ccp1 over-expression promoted cell proliferation in both the presence and absence of serum.

### FGF2 treatment increases the proliferative effect of ccp1

**Figure 3:**
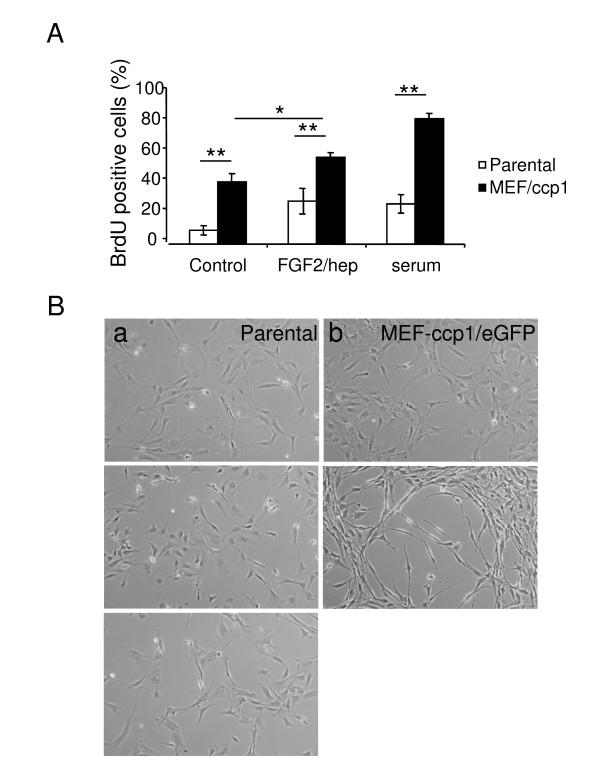
**Effects of mitogen stimulation on ccp1-induced cell proliferation and cell shape changes**. MEF expressing ccp1 and parental MEF were cultured in absence of serum overnight and treated with FGF2 and heparin or 10% serum, for 24 h. (A) One hour pulse label of BrdU was performed and BrdU incorporation was calculated as described before (**p < 0.005). (B) Parental MEF and MEF stably expressing ccp1 were cultured in absence of serum overnight (a-b) and treated with FGF2 and heparin (c-d) or serum (e-f). Upon FGF2 stimulation for 24 h, MEF stably expressing ccp1 showed more drastic changes in cell shape. Similar changes were observed in the presence of serum.

Ccp1 has been previously identified as a downstream gene of FGF2 [1]. Therefore, to further investigate whether ccp1 is an effecter of FGF2 mediating its signal in cell proliferation, control parental MEF expressing GFP and MEF expressing ccp1/eGFP-N were starved overnight and treated with 50 ng/ml FGF2 or with 10% serum (Figure 3A). BrdU was added to the culture for 24 h. In the control where no ligand or serum was added, MEF expressing ccp1 showed increase in BrdU incorporation in comparison to parental MEF. Further increase in BrdU incorporation was observed in cells treated with FGF2. However in MEF expressing ccp1, the level of increase by FGF2 did not reach the level achieved by serum. After stimulation with serum, more cells in S phase were observed in comparison to the FGF2 treated cells in MEF expressing ccp1. Parental MEF showed incorporation of BrdU in 28.5% and 26.8% of cells when treated with FGF2 and serum, respectively. In contrast, MEF expressing ccp1 showed the BrdU incorporation in 57.4% and 83.1% of cells in FGF2 and serum, respectively. These data suggested that effects of ccp1 in cell proliferation were enhanced by FGF2, and that other mitogens present in the serum are likely to also enhance these effects.

We showed that ccp1 expression in MEF and SK-N-SH cells caused a change in cell morphology (Figure 1C). Here we addressed whether FGF2 played a role in ccp1-induced changes in cell morphology, compared to the effect of serum. Control parental MEF and MEF expressing ccp1/eGFP-N were starved overnight and treated with either 50 ng/ml FGF2 in the presence of 10 μg/ml heparin, or serum, for 24 h (Figure 3B). The morphological changes observed in ccp1-expressing cells became more prominent upon stimulation with FGF2 (Figure 3B, c-d) and changes were similar upon treatment with serum (Figure 3B, e-f).

### BrdU incorporation is inhibited when ccp1 expression is knocked down by RNAi

**Figure 4:**
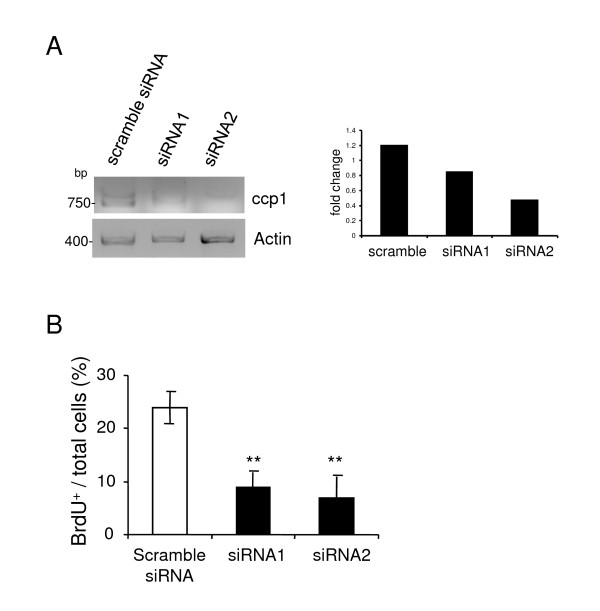
**Cell proliferation was inhibited when ccp1 expression was knocked down in NIH3T3 cells**. (A) Ccp1 expression was knocked down in NIH3T3 cells using specific ccp1-siRNA (siRNA1 and siRNA2). Ccp1 expression after silencing was assessed by RT-PCR. Actin amplification was performed to show equal sample loading. Densitometry analysis showed the fold changes. (B) BrdU assay performed in absence of serum showed that cell proliferation in cells knocked down with ccp1 decreased compared to the scramble siRNA-treated cells (n = 3, ** = p < 0.005). Cells transfected with Scramble siRNA did not show significant reduction in cell proliferation.

To further confirm the role of ccp1 in cell proliferation, RNAi was performed. NIH3T3 cells were transfected with small interfering RNAs (siRNAs) specific for ccp1 (siRNA1 and siRNA2) and a scramble siRNA as a control. After 24 h, mRNA levels of ccp1 were measured by semi-quantitative RT-PCR. Ccp1 expression was reduced in cells transfected with the siRNA1 and siRNA2 comparing to scramble siRNA and this was confirmed by densitometry analysis (Figure 4A right panel). Next, BrdU assays were performed in cells transfected with ccp1 siRNAs (Figure 4B). Upon knockdown of ccp1 expression, cells showed a decrease in BrdU incorporation compared to the scramble siRNA-treated cells. Therefore, these data further confirmed the role of ccp1 in promoting cell proliferation.

### Ccp1 stable expression protects cells from apoptosis

**Figure 5:**
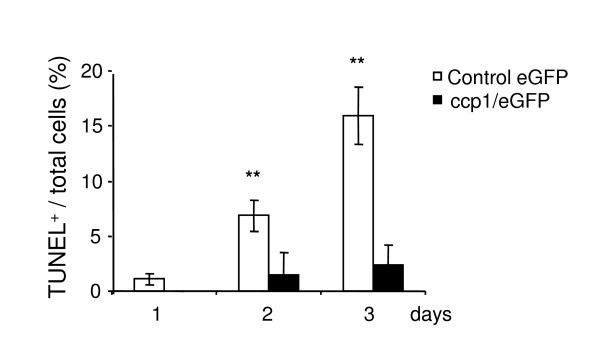
**Stable expression of the ccp1 protein reduced apoptosis in the absence of serum**. TUNEL assay was performed in SK-N-SH cells stably expressing ccp1/eGFP protein and eGFP alone as a control. Cells were cultured in the absence of serum for three days. The number of cells that went through apoptosis increased in the control, up to 15.9 ± 2.6% of total number of cells visualized by DAPI on the third day. In contrast, apoptotic cells in cells expressing ccp1/eGFP protein remained low, up to 2.3 ± 1.8% (n = 3) (**p < 0.005).

Apoptosis or programmed cell death is an important physiological process that contributes to final cell number in the tissue. In order to determine whether apoptosis was involved in the increase in cell number upon over-expression of ccp1 (Figure 2), TUNEL assay was performed in SK-N-SH cells (Figure 5). After 24 h of plating, cells were cultured in the absence of serum for 1, 2, and 3 days before TUNEL assay was performed. At day 1, no apoptotic cells were detectable in SK-N-SH cells expressing ccp1. However a major effect was evident at day 2 and 3, where SK-N-SH cells expressing ccp1 showed a 5 and 7 fold decrease in apoptotic cells in comparison with SK-N-SH cells expressing eGFP, respectively. These data suggest that ccp1 plays a role in suppressing cell death.

### Effects of MEK inhibitor, U0126, in cell proliferation upon ccp1 over-expression

**Figure 6:**
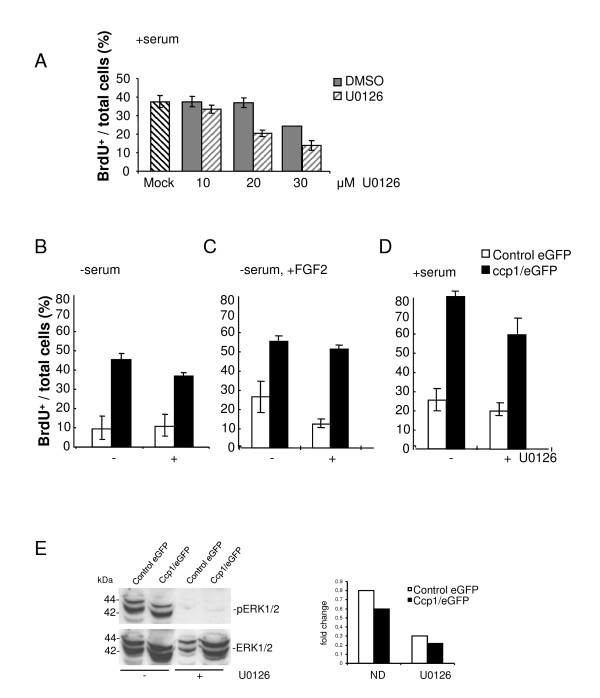
**Cell proliferation promoted by ccp1 was partially reduced by addition of MEK inhibitor **. (A) MEF cells were treated with various concentrations of U0126 in the presence of serum for 24 h and BrdU assay was performed. The concentration of 20 μM was chosen. (B) Cells expressing ccp1 were serum starved overnight, treated with 20 μM of U0126 and BrdU assay was performed. (C) Cells expressing ccp1 were serum starved overnight, treated with 50 ng/ml FGF2 and 10 μg/ml heparin, 20 μM of U0126 or combinations as indicated for 24 h and BrdU assay was performed. (D) Cells expressing ccp1 were treated with 10% serum, 20 μM of U0126 or combinations as before. (E) Control MEF expressing eGFP and MEF expressing ccp1 were treated with 20 μM of U0126 for 24 h and Western blotting was performed to detect the level of p-ERK. Densitometry analysis showed the fold changes in p-ERK1/2.

The MAPK signaling is involved in cell proliferation and is one of the core signaling pathways of FGF [11]. Therefore, to identify the signaling mechanism of cell proliferation promoted by ccp1, MAPK signaling was investigated in MEF expressing ccp1/eGFP-N. In order to inhibit this signaling pathway and see its effects in cell proliferation, the MEK inhibitor, U0126, was used in the BrdU assays. First, the optimal concentration of U0126 was determined in the culture system used (Figure 6A). Treatment with 10 μM U0126 resulted in a 10.4% reduction in cell proliferation in comparison to untreated cells. 20 μM U0126 caused a 45.3% decrease in proliferation in comparison to the untreated cells. DMSO alone did not show any effect at this concentration. Because of DMSO toxicity at 30 μM, we selected the concentration of 20 μM.

Control MEF expressing eGFP and MEF expressing ccp1/eGFP-N were cultured overnight in the absence of serum, in the presence and absence of 20 μM U0126 (Figure 6B). The effectiveness of U0126 was addressed in these cells by Western blotting and densitometry analysis, which confirmed reduced phosphorylated ERK upon treatment (Figure 6E). In the absence of U0126, an increase in BrdU incorporation was detected in the MEF stably expressing ccp1 compared to the control MEF (Figure 6B). Upon treatment by U0126, although BrdU incorporation remained similar in control MEF, a decrease was observed in MEF expressing ccp1. The effects of MEK inhibition was also analysed in cells treated with FGF2 and 10 μg/ml heparin (Figure 6C), as well as with 10% serum (Figure 6D). BrdU incorporation was partially inhibited in control MEF and in MEF expressing ccp1. This indicates that MAPK may play a partial role in cell proliferation promoted by ccp1 over-expression, however there are signaling pathways other than MAPK that are likely to be also involved in this process.

### MAPK signaling upon ccp1 over-expression

**Figure 7:**
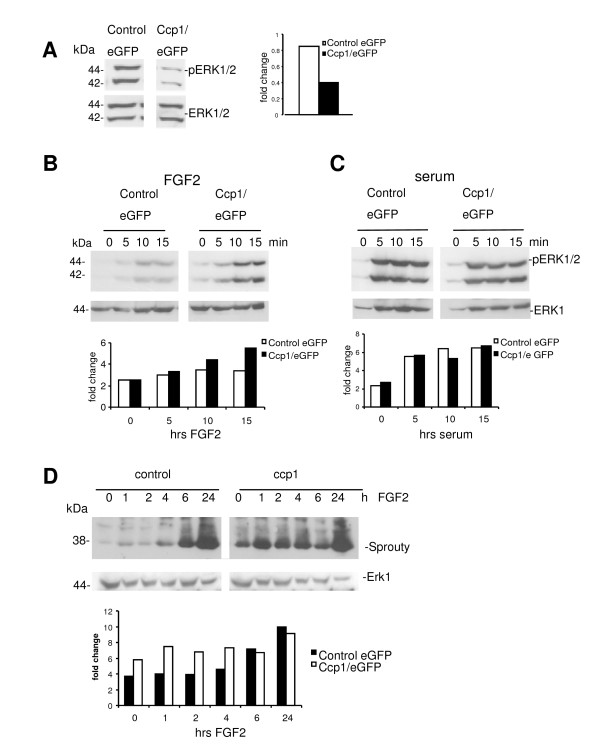
**p-ERK and SPRY levels in ccp1 over-expressing cell**. (A) p-ERK was decreased in ccp1-expressing MEF in the steady state of culture under serum. (B) Cells were starved and treated with FGF2 for time indicated. Western Blotting was performed to examine levels of p-ERK. (C) A similar experiment was carried out for a treatment with serum. (D) MEF expressing ccp1 were cultured in absence of serum for 24 h and treated with 50 ng/ml FGF2 in the presence of 10 ug/ml heparin for time indicated. Levels of SPRY were analysed by Western Blotting. 7.5% SDS-PAGE gels were used. Densitometry analysis showed the fold changes for each blot, showing changes in p-ERK1/2 (A-C) and Spry2 (D).

We have also performed Western blotting to analyze activation of MAPK signaling using anti p-ERK antibody and densitometry analysis. Unexpectedly, ccp1-expressing MEF showed a decrease in ERK phosphorylation in the steady state culture under serum (Figure 7A). To characterize further the nature of ERK phosphorylation in ccp1-over-expressing cells, levels of p-ERK were examined upon either FGF2 or serum stimulation over time. Cells were starved overnight and treated with 50 ng/ml FGF2 in the presence of 10 μg/ml heparin for a short time period of up to 15 min (Figure 7B). Control MEF showed a gradual activation of ERK as early as 5 min of stimulation. In contrast, a similar but more significant increase was observed in the cells expressing ccp1 as early as 5 min. In contrast, no obvious difference in the level of p-ERK was detected between the control and the ccp1 expressing cells in the presence of 10% serum (Figure 7C). These data suggests that ccp1 expression may modulate ERK phosphorylation upon treatment with FGF2.

MAPK signaling events are regulated by a negative-feedback loop through Sprouty (SPRY) proteins [17]. SPRY expression is induced by MAPK signaling upon growth factor stimulation, such as FGF2 [18]. Therefore we addressed whether ccp1 could induce SPRY feedback regulatory activity. Levels of SPRY were analysed by Western blotting in control and ccp1 expressing MEF using a pan-SPRY antibody followed by densitometry analysis (Figure 7D). MEF expressing ccp1 were cultured in the absence of serum overnight and treated with 50 ng/ml FGF2 in the presence of 10 μg/ml heparin for up to 24 h. In the control MEF, the level of SPRY was low at time 0 and gradually increased upon FGF2 stimulation over the time. In contrast, in the MEF expressing ccp1, the level of SPRY was higher than in the control MEF from the time point 0 and reached the maximum level observed in the presence of FGF2 at 6 hours.

## Discussion

In this study, we have investigated the potential role of ccp1, with a hypothesis that ccp1 may be a downstream effecter of FGF2, promoting cell proliferation and protecting from apoptosis. We show here that ccp1, a gene expressed in embryonic and adult brain [1], may regulate cell morphology, proliferation and programmed death in normal fibroblast and in neuroblastoma cells.

We have demonstrated that morphological transformation occurred in MEF and SK-N-SH cells under stable over-expression of ccp1 (Figure 1). This effect was observed both in the presence or absence of FGF2 or serum (Figure 3). A long-term culture, for example, of neurite outgrowth or growth of cells in soft agar, may further clarify the effect of ccp1 in morphological transformation in the presence or absence of FGF2 in the future. Growth curve and BrdU incorporation in the presence of serum showed that ccp1 expression is able to promote proliferation up to 5 days in culture (Figure 2). Similar results were observed in the absence of serum, suggesting that ccp1 is able to induce proliferation without mitogen stimulation (Figure 2 and 3). Reduction of ccp1 level by RNAi dramatically reduced SK-N-SH cell proliferation, providing further evidence that ccp1 can induce cell proliferation (Figure 4). In addition, we have showed that ccp1 plays a role in suppressing cell death (Figure 5). Whether extrinsic from intrinsic pathways are involved in the suppression of apoptosis by ccp1 is unknown and will need further investigation.

Although both FGF2 and serum treatment enhanced the increase in cell proliferation upon ccp1 over-expression, the effect of FGF2 did not reach that of the serum (Figure 3 and 6). Therefore it is likely that other mitogenic factors present in the serum can also enhance this ccp1 activity. In addition, it was shown that in the absence of serum, ccp1-induced proliferation was partially inhibited by U0126 (Figure 6B). In contrast, in cells treated with FGF2, the inhibition was less prominent in MEF expressing ccp1 than control MEF (Figure 6C, D). This indicates that MAPK may play only a partial role in cell proliferation promoted by ccp1 over-expression, and that there are signaling pathways other than MAPK that are likely to be also involved in this process. For example, ccp1 activity could be regulated by several signaling pathways, such as the PI3K/AKT pathway. The use of inhibitors of the AKT pathway, such as an mTor inhibitor, rapamycin, may be useful in the future to clarify this point.

It is still unclear how ccp1-induced proliferation is enhanced by FGF2 or serum, in particular, either as a consequence of an increased expression of endogenous ccp1 induced by mitogens such as FGF2 [1], or functional enhancement of ccp1 activity by these factors.

Ccp1 expression was able to increase ERK phosphorylation immediately after the treatment with FGF2 (Figure 7B). This could be the bases of ccp1-induced cell proliferation observed in Figure 6. However, ccp1-expressing MEF showed a decrease in ERK phosphorylation in the steady state culture under serum (Figure 7A). As the decrease in MAPK signaling could be due to the presence of the feedback loop such as Sprouty (SPRY) proteins [17], we analysed the SPRY levels (Figure 7D). In MEF expressing ccp1, the level of SPRY was already higher than in the control MEF at the time point 0, however at 6 hours, it reached the maximum level observed in the presence of FGF2. This may indicate that ccp1 expression accelerated the induction of SPRY level, possibly due to activation of MAPK signaling. However it is remains unclear, why pERK was suppressed upon ccp1 over-expression in the steady state of culture. On the other hand, maintained increase in SPRY levels in ccp1-overexpressing cells (Figure 7D) may explain the decrease in cell growth observed in the absence of serum at 4 days of treatment (Figure 2C).

Aberrant activation of FGFs and their receptors lead to several pathologies, including cancer [19]. Study of ccp1 function in promoting proliferation and suppressing cell death would be interesting in aiming a better understanding of tumor formation. Further experiments using knockdown system of ccp1 are necessary to address the requirement of ccp1 in mediating FGF signaling in cell proliferation and apoptosis, possibly using multiple cell lines. Although unlikely, a potential cannot be excluded that the siRNA regulates the protein level of ccp1 differently from that of the mRNA. This has to be addressed in the future experiments.

## Conclusions

This study has shown that ccp1 regulates cell proliferation and cell death. Although FGF2 enhanced the effects of ccp1, other mitogenic factors such as MAPK, are likely taking part in enhancing the effects of ccp1.

## Abbreviations

BrdU: 5-Bromo 2-Deoxyuridine; Ccp1: Coiled Coil protein 1; CNC: Cortical Neuron Culture; eGFP: Enhanced Green Fluorescent Protein; ERK: Extracellular-signal Regulated Kinase; FGF: Fibroblast Growth Factor; GAPDH: Glyceraldehydes-3-Phosphate Dehydrogenase; MEF: mouse embryonic fibroblast; MEK: MAPK or ERK Kinase; mRNA: messenger Ribonucleic Acid; MAPK: Mitogen-Activated Protein Kinase.

## Competing interests

The authors declare that they have no competing interests.

## Authors' contributions

FP and TI designed the research and analysed the data. FP and RT performed the experiments. FP and TI wrote the manuscript. GI helped to design the research. All authors commented on the manuscript.

## Pre-publication history

The pre-publication history for this paper can be accessed here:

http://www.biomedcentral.com/1471-2407/10/657/prepub
